# Immunopathological outcomes are isolate dependent in chronic *Mycobacterium avium* complex pulmonary disease

**DOI:** 10.1242/dmm.052671

**Published:** 2026-01-30

**Authors:** Timothy D. Shaw, Ha Lam, Taru S. Dutt, Camron M. Pearce, Ilham Alshiraihi, Andres Obregon-Henao, Marcella Henao-Tamayo, Sara E. Maloney Norcross, Bernd Meibohm, Mary Jackson, Mercedes Gonzalez-Juarrero

**Affiliations:** ^1^ Wellcome-Wolfson Institute for Experimental Medicine, School of Medicine, Dentistry and Biomedical Sciences, Queen's University Belfast, Belfast BT9 7BL, UK; ^2^ Mycobacteria Research Laboratories, Department of Microbiology, Immunology and Pathology, College of Veterinary Medicine and Biomedical Sciences, Colorado State University, Fort Collins, CO 80523-1682, USA; ^3^ Technology Advancement and Commercialization, RTI International, Research Triangle Park, NC 27709, USA; ^4^ Department of Pharmaceutical Sciences, University of Tennessee Health Science Center, Memphis, TN 38163, USA

**Keywords:** Non-tuberculous mycobacteria, *Mycobacterium avium* complex, Animal model, Histopathology, Immunology

## Abstract

Novel treatment strategies are urgently needed to combat *Mycobacterium avium* complex (MAC) pulmonary disease (PD). Animal models are important for screening therapeutic strategies, but their ability to reproduce human-like immunopathology and impaired respiratory function is poorly characterised. We modelled chronic lung infection in BALB/c mice over 20 weeks with three isolates of MAC (MAC101, MAC104 and MAC2285R) to compare bacterial growth, histological injury, immune cellular dynamics and respiratory function. We found that MAC101 caused a proliferative infection over 20 weeks, associated with a strong adaptive response, progressive granulomatous inflammation and increasing respiratory effort. For MAC104, lung bacterial burden rose initially but fell after week 12, accompanied by increased regulatory T-cell response and stabilisation of pathological and respiratory changes. By contrast, MAC2285R caused a low-virulence, non-proliferative infection associated with a strong myeloid cell response, modest histopathological change and increased respiratory effort. Immune cell dynamics in chronic murine MAC-PD correlate with bacterial burden and pathology and are strongly MAC-isolate dependent. These findings provide a spectrum of quantifiable and clinically relevant disease outcomes to facilitate the preclinical screening of novel antimicrobial and host-directed therapies for MAC-PD.

## Teaser

**Summary:** Clinically relevant outcomes in chronic *Mycobacterium avium* complex pulmonary disease (including bacterial burden, histological injury, immune cellular dynamics and respiratory function) are variably dependent on the bacterial isolate.

## INTRODUCTION

The emergence of *Mycobacterium avium* complex (MAC) as a cause of treatment-refractory lung infection is well described (Diel et al., 2017; Patterson et al., 2024). Chronic MAC pulmonary disease (MAC-PD) is characterised by tissue-destructive granulomatous inflammation and reduced quality of life due to impaired respiratory function (Choi et al., 2021; Park et al., 2023). Novel therapeutic strategies are urgently needed, particularly to clear the infection and attenuate immune-mediated lung injury. However, MAC-PD is a heterogeneous disorder, and the spectrum of immunopathological outcomes seen in patients is poorly understood (Choi et al., 2021). Moreover, there is a lack of well-characterised, clinically relevant animal models capable of replicating the continuum of human disease to screen candidate therapies (Daniel-Wayman et al., 2019).

The BALB/c mouse has become the primary strain used in preclinical testing of novel therapeutic strategies for MAC-PD (Andréjak et al., 2015; Shaw et al., 2024). It demonstrated superior susceptibility to MAC pulmonary infection and treatment response to other mouse strains in a comparative study (Andréjak et al., 2015). Similarly, MAC101, a blood isolate originally derived from clinically disseminated disease, has become the standard bacterial reference strain used for *in vitro* and *in vivo* susceptibility testing of antimicrobial therapies (Woods et al., 2011). BALB/c mice infected with MAC101 generate a proliferative lung infection – characterised by a higher rate of bacterial replication than bacterial death, causing a net increase in bacterial burden – with inflammatory foci observable on gross examination of resected lungs (Andréjak et al., 2015). Lung bacterial burden appears responsive to antimicrobial therapy (Lanoix et al., 2020), but degrees of pathology and responsiveness to antimicrobials have not been quantified in the MAC101-infected BALB/c mouse. Moreover, other clinically relevant outcomes, such as lung immune cell profiling and respiratory function, have not been described in this model.

Therefore, we sought to characterise MAC-PD in the BALB/c mouse with quantifiable, clinically relevant features that more closely replicate the spectrum of human disease. We infected BALB/c mice intrapulmonary aerosols with one of three clinically isolated MAC isolates: MAC101, MAC104 (a blood isolate from clinically disseminated disease) and MAC2285R (a respiratory isolate from aggressive pulmonary disease). We then examined for differences and correlations in bacterial growth, lung pathology, immune cellular dynamics and respiratory function in chronic murine MAC-PD over 20 weeks’ infection.

## RESULTS

### Bacterial and pathological outcomes in chronic *M. avium* infection are isolate dependent

**Fig. 1.**
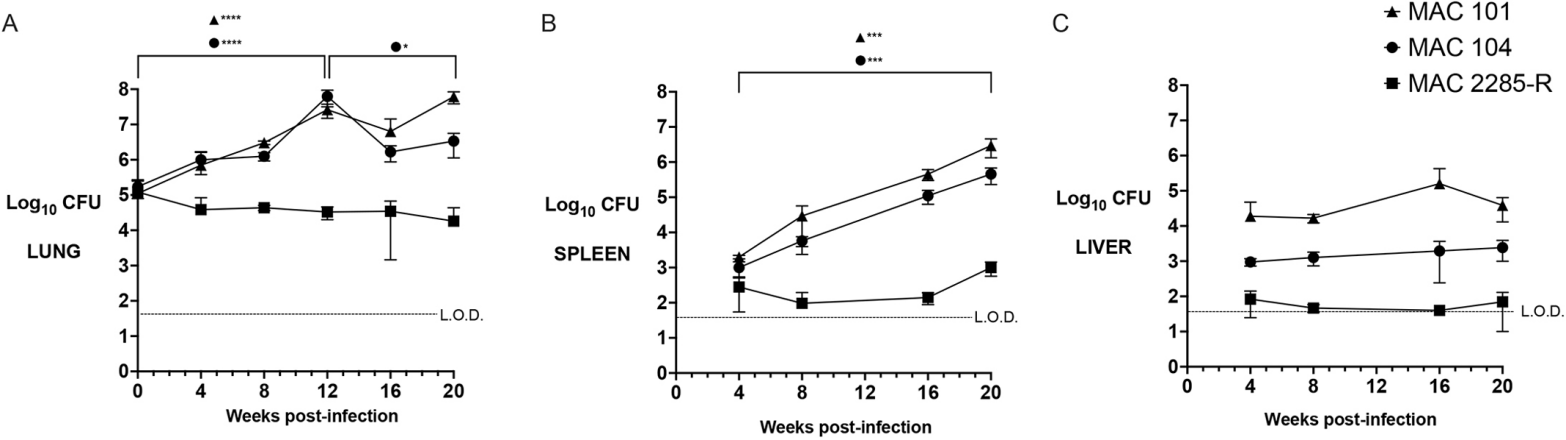
**Comparative time course infection of three isolates of *Mycobacterium avium* complex (MAC) in BALB/c mice.** (A-C) Lung (A), spleen (B) and liver (C) bacterial burden [*y*-axis, log_10_ colony-forming units (CFU)] in BALB/c mice infected by intrapulmonary aerosol with high-dose inoculum (10^5^ CFU) per lung was measured over 20 weeks' infection with MAC101, MAC104 or MAC2285R. MAC101 and MAC104 proliferated and disseminated at a significantly higher rates than MAC2285R. Data combined from two independent studies, *n*=3-8 per time point. Data are mean±s.d. for each time point and were analysed by one-way ANOVA and Tukey's multiple comparison test using GraphPad Prism v.10. *P<0.05, ***P<0.001, ****P<0.0001. L.O.D, limit of detection.

Lung bacterial burden rose in MAC101- and MAC104-infected mice at similar rates for the first 12 weeks post-infection, peaking at mean 7.4 log_10_ and 7.8 log_10_ colony-forming units (CFU), respectively (Fig. 1A, *P*<0.0001 compared to week 0). MAC104 lung burden then fell at week 16 (to mean 6.2 log_10_ CFU, *P*<0.0001) and remained stable at week 20 (6.5 log_10_ CFU, *P*=0.36 compared to week 16), whereas MAC101 entered a stable, plateau phase (at 7.8 log_10_ CFU, *P*=0.06). By contrast, MAC2275R lung burden fell gradually over time from baseline over 20 weeks, with a net decrease of 0.8 log_10_ CFU (from 5.1 log_10_ CFU to 4.3 log_10_ CFU), although this did not reach statistical significance (*P*=0.22). MAC101 and MAC104 CFU were detectable in spleen (Fig. 1B) from week 4 and rose by >3 logfold over 20 weeks (*P*<0.001 for both isolates). Splenic CFU of MAC2285R were also detectable after 4 weeks, although there was no significant change over 20 weeks (*P*=0.13). Hepatic CFU were detectable after 4 weeks in mice infected with all three isolates (Fig. 1C). MAC101 infection resulted in higher liver burden than did MAC104 and MAC2285R infection over the 20-week study (mean 4.5 log_10_ CFU versus 3.3 log_10_ CFU and 1.6 log_10_ CFU, respectively; *P*<0.01 for both comparisons).

**Fig. 2.**
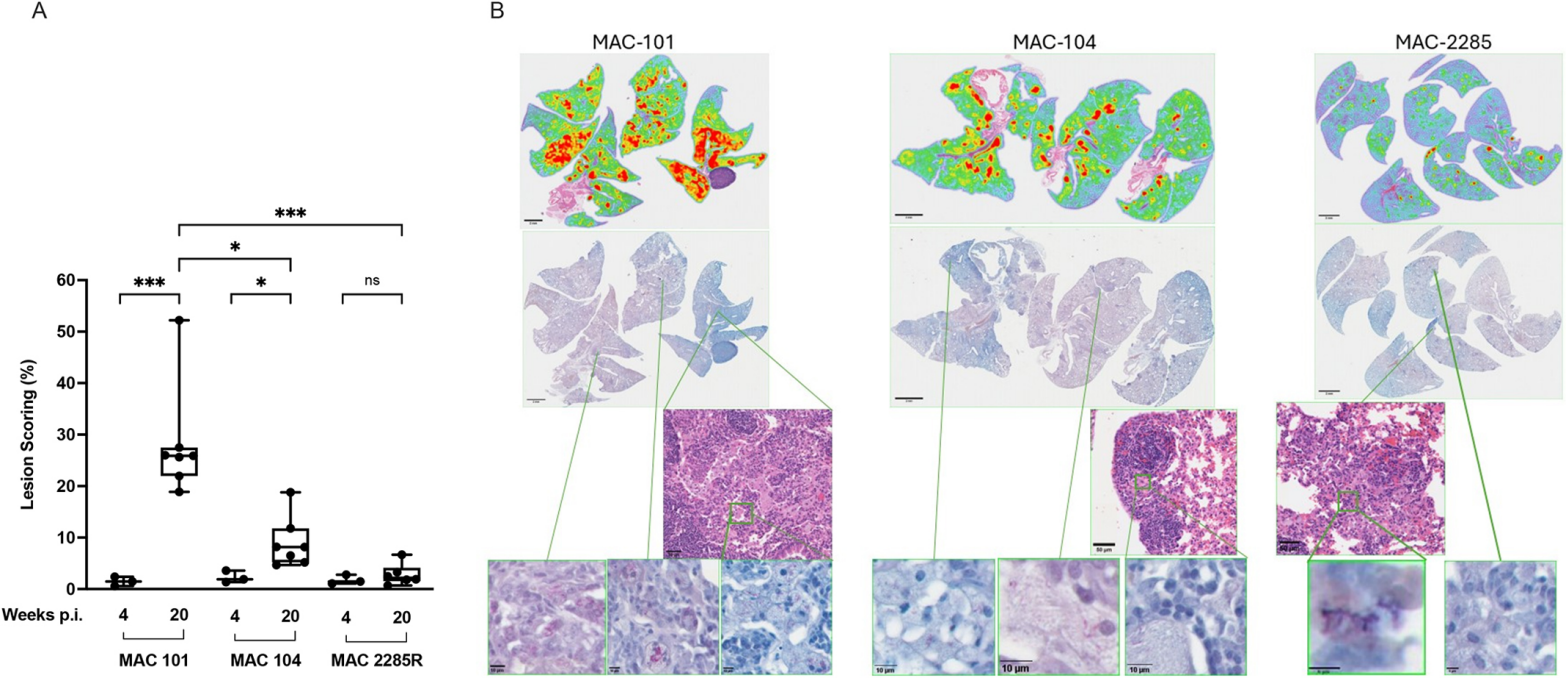
**Comparative histopathological analysis of BALB/c mice with MAC pulmonary disease (MAC-PD).** Lungs were harvested at weeks 4 and 20 post-infection from BALB/c mice infected with MAC101, MAC104 or MAC2285R. (A) Lesion scores were calculated as the proportion of infected area over the total lung area per animal (*n*=3-7). Mice exhibited progressive increases in mean lesion scoring between weeks 4 and 20 post-infection when infected with MAC101 or MAC104 (*P*<0.001 and *P*<0.05, respectively). By contrast, lesion scoring remained unchanged in mice with MAC2285R infection over the study period (*P*=0.34). By week 20, MAC101 infection resulted in higher lesion scores (mean, 28.3%) than MAC104 (mean, 9.0%; *P*<0.05) and MAC2285R (mean, 2.8%; *P*<0.001). (B) Representative slices of lungs from mice infected with each isolate are displayed, together with heat mapping of inflammation (green) and granulomas (red). Data combined from two independent studies, *n*=3-7 per time point. Data were analysed by one-way ANOVA with Tukey's multiple comparison test for comparison between MAC isolates, or by unpaired two-tailed *t*-test for comparison between time points with the same isolate. *P<0.05, ***P<0.001; n.s., not significant. Scale bars: 2 mm (top panels), 50 μm (middle panels), 10 μm (bottom panels).

Histopathology with quantification of lesion score and presence of acid-fast positive bacilli (AFB) was evaluated in lung sections from BALB/c mice infected with MAC101, MAC104 or MAC2285R at 4-week intervals from weeks 4 to 20 post-infection (Fig. S1). Comparisons of lesion scores were performed between weeks 4 and 20 for each MAC isolate, as well as between MAC isolates at week 20 (Fig. 2A,B). Areas of inflammation – defined as evolving infiltrates of neutrophils, monocyte/macrophages and lymphocytes – were observed with thickening of the parenchyma, displayed in green in Fig. 2A heat maps. Granulomas, displayed as red in Fig. 2A heat maps, consisted of macrophages (some with abundant cytoplasmic foamy vacuolation, also referred to as foamy) and infiltration of lymphocytes. Histopathological analysis of Haematoxylin and Eosin (H&E)-stained lung sections revealed mild pathology after 4 weeks’ infection for mice infected with all three MAC isolates: mean lesion scoring was 1.5% (±0.9 s.d.) for MAC101, 2.3% (±1.2 s.d.) for MAC104 and 1.8% (±0.9 s.d.) for MAC2285R, with no significant difference between isolates. However, over the study period, mice with MAC101 infection exhibited progressively more severe histological injury (Fig. S1), with mean lesion scoring of 28.3% (±10.9 s.d.) at week 20 post-infection (*P*<0.001, compared to week 4) (Fig. 2A). Similarly, lesion scoring rose for MAC104-infected mice, although they did not reach the same level of severity, with a mean score of 9.0% (±4.9 s.d.) after 20 weeks (*P*<0.05). By contrast, the degree of histological injury remained static in mice with MAC2285R infection, with a mean score of 2.8% (±2.1 s.d.) after 20 weeks (*P*=0.34).

### Immune cellular dynamics in chronic *M. avium* infection are isolate dependent

We noted that lung bacterial burden between the three MAC isolates diverged between weeks 12 and 20, in that MAC101 CFU remained high without significant change, MAC104 CFU fell then plateaued, and MAC2285 CFU continued to fall gradually (Fig. 1). Therefore, in the repeat study, we examined whether each of these isolates elicit different immune response in lungs and, if so, which cells are correlated with clearance versus increase in bacterial burden in lungs at week 16 post-infection.

**Fig. 3.**
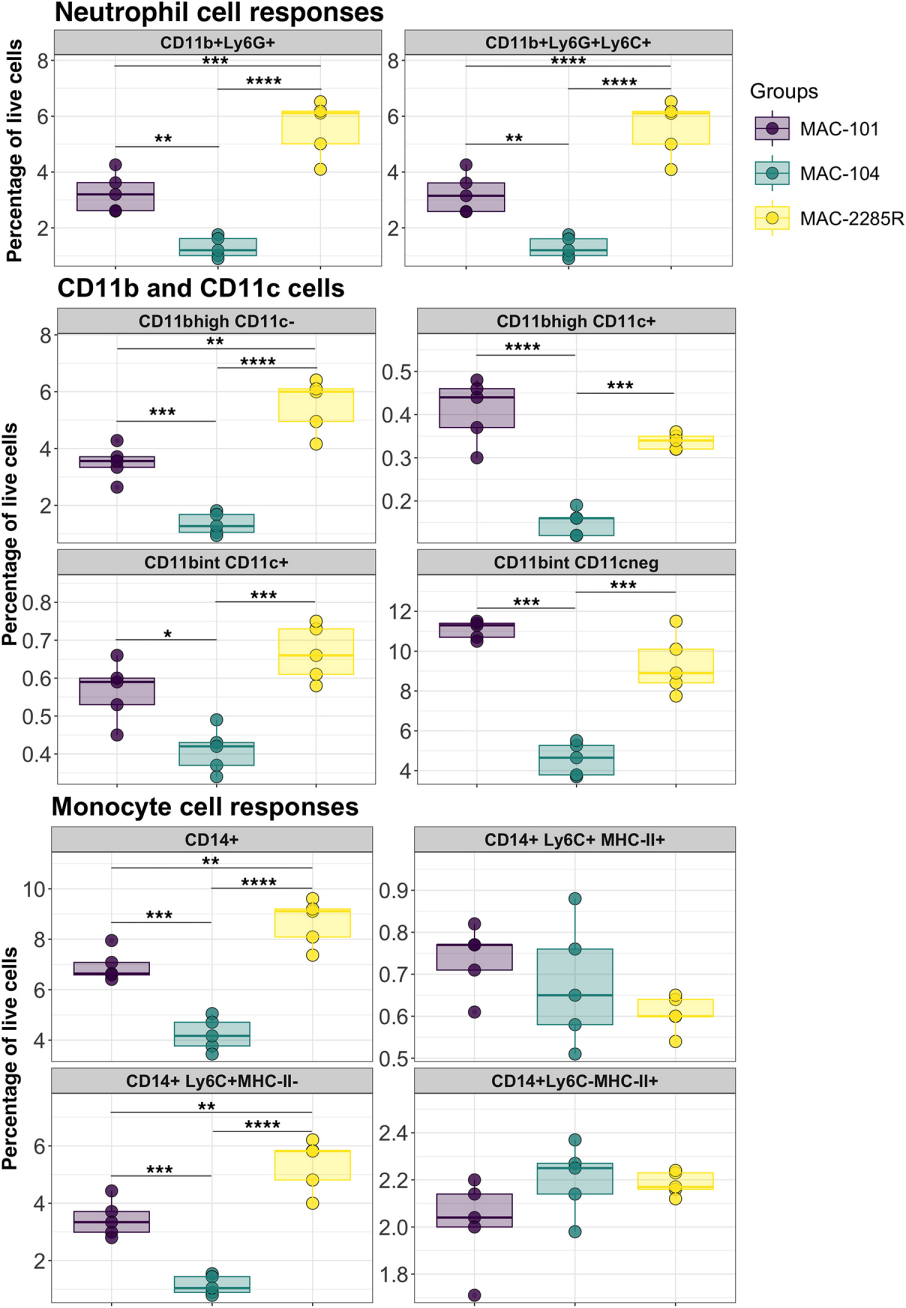
**Comparative flow analysis on myeloid cell response in lungs of BALB/c mice with chronic MAC infection.** Myeloid cell responses in the lungs were analysed using spectral flow cytometry. Lung tissues were collected 16 weeks post-infection from mice infected with MAC101, MAC104 or MAC2285R (*n*=5). Data are representative of one study with *n*=4, presented as individual points with mean and interquartile range. Statistical analysis was performed using one-way ANOVA with Tukey HSD. *P<0.05, **P<0.01, ***P<0.001, ****P<0.0001.

**Fig. 4.**
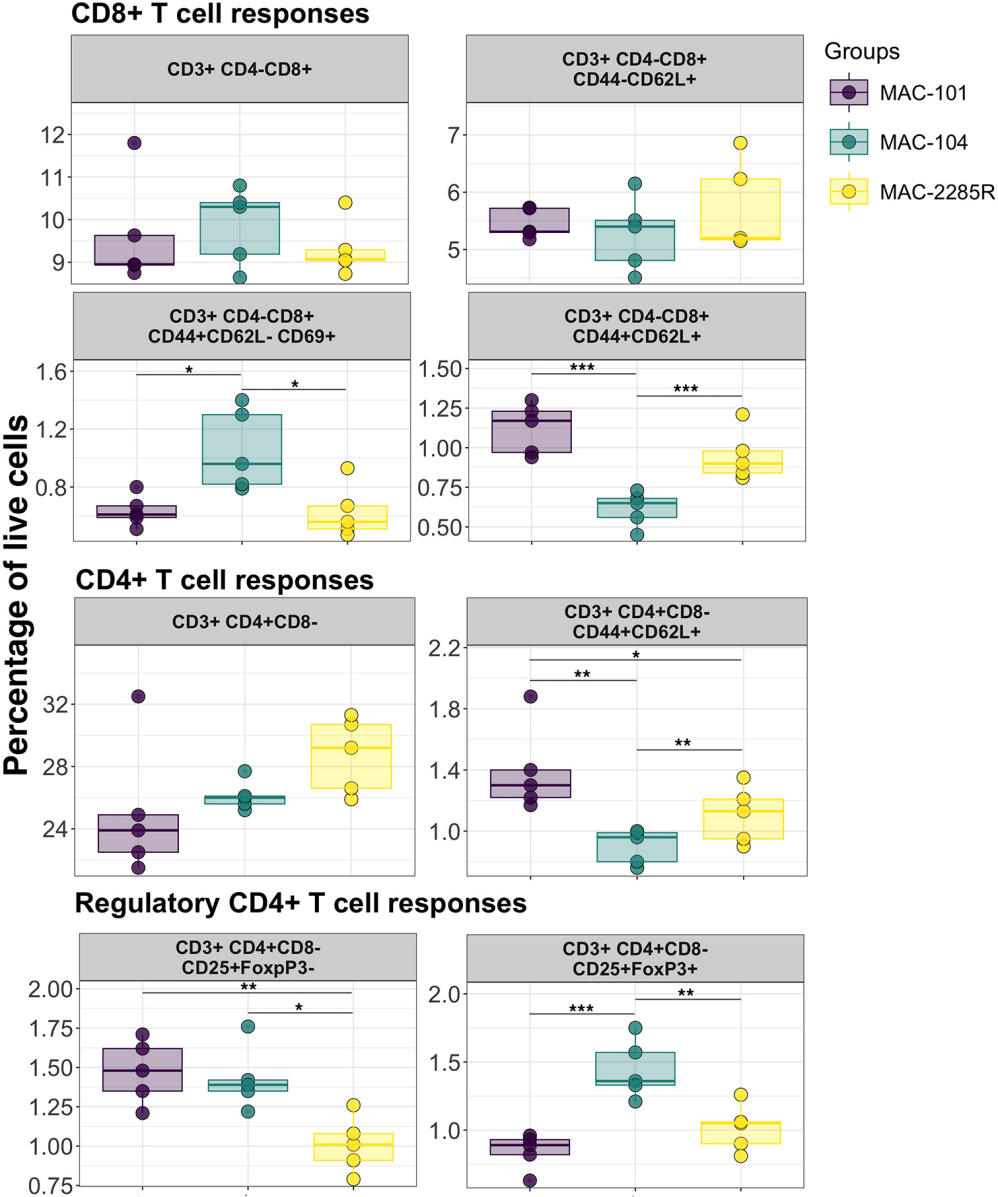
**Comparative flow analysis of T-cell responses in lungs of BALB/c mice with chronic MAC infection.** Lungs were harvested 16 weeks post-infection from mice infected with MAC101, MAC104 or MAC2285R (*n*=5). Flow cytometry was performed to evaluate the frequency and activation state of T-cell populations, including CD4^+^ and CD8^+^ T cells, as well as their expression of key markers associated with activation, exhaustion or differentiation. Data are representative of one study with *n*=4, presented as individual points with mean and interquartile range. Statistical analysis was performed using one-way ANOVA with Tukey HSD. *P<0.05, **P<0.01, ***P<0.001.

Myeloid cell responses were significantly elevated in mice infected with MAC2285R followed by MAC101 and then MAC104 (Fig. 3). Specifically, neutrophils (CD11b^+^Ly6G^+^Ly6C^+^), CD11b^int^ dendritic cells (CD11b^int^CD11c^+^) and monocytes (CD14^+^ and CD14^+^Ly6C^+^) were significantly higher in the lungs of MAC2285R-infected mice than those in the lungs of MAC101- and MAC104-infected mice (*P*<0.01 and *P*<0.0001, respectively). Interestingly, CD11b^high^ dendritic cells (CD11b^high^CD11c^+^) (Li et al., 2008) exhibited a distinct pattern, with the highest frequency observed in MAC101-infected lungs, followed by MAC2285R-infected lungs and the lowest levels in MAC104-infected lungs (*P*<0.001). In the context of T-cell responses, we found isolate-specific differences as well (Fig. 4). The levels of activated CD8^+^ T cells (CD3^+^CD4^−^CD8^+^CD44^+^CD62L^−^CD69^+^) were highest in MAC104 infection (*P*<0.05), whereas MAC101 and MAC2285 infection yielded comparable numbers of activated CD8^+^ T cells. Furthermore, both CD8^+^ and CD4^+^ central memory T-cell populations (CD3^+^CD8^+^CD44^+^CD62L^+^ and CD3^+^CD4^+^CD44^+^CD62L^+^, respectively) were detected in the lungs, with the highest levels observed in MAC101-infected mice (*P*<0.05), followed by MAC2285R- and MAC104-infected mice. Interestingly, the levels of CD4^+^ regulatory T cells (Tregs; CD3^+^CD4^+^CD25^+^Foxp3^+^) presented an opposite trend. The highest numbers of Tregs were observed in mice with MAC104 infection (*P*<0.01); those with MAC101 and MAC2285R infections had significantly lower Treg levels, which were comparable between the two isolates. Overall, our findings highlight the isolate-specific immune response profiles elicited by infection with different MAC isolates: MAC2285R drives robust recruitment of neutrophils, macrophages and monocytes, while MAC101 elicits the highest dendritic cell and central memory T-cell responses. Conversely, MAC104 predominantly induces a strong Treg response, suggesting unique immunomodulatory mechanisms across MAC isolates.

### Isolate-specific lung immune signatures correlate with bacterial burden and pathology

**Fig. 5.**
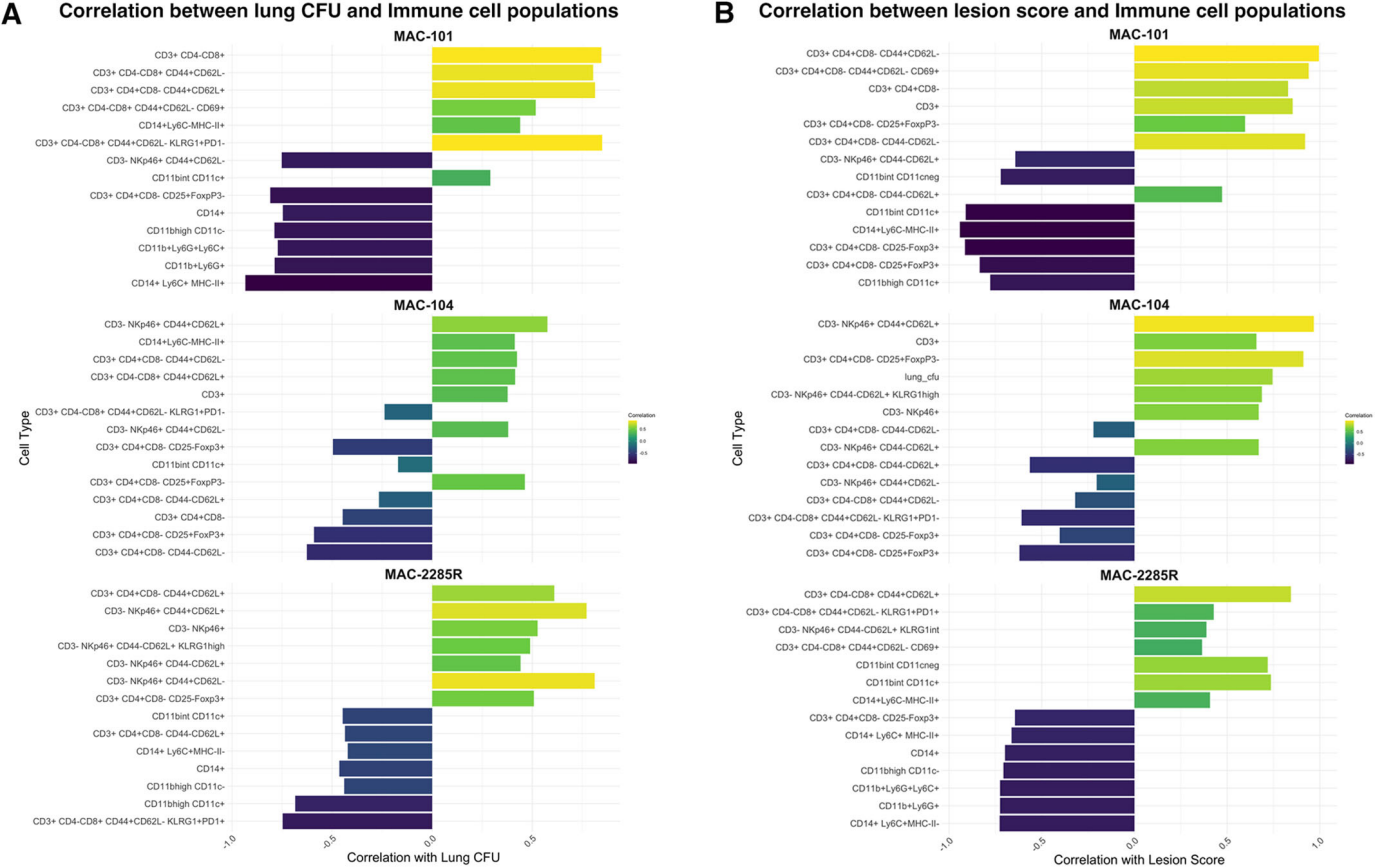
**The top seven positively and negatively correlated subsets for each MAC isolate by lung CFU and lesion score.** (A,B) Activated CD4^+^ and CD8^+^ T cells consistently exhibited positive correlations with both lung CFU (A) and lesion scores (B) for all three MAC isolates. By contrast, myeloid populations were consistently significantly negatively correlated with both lung CFU and lesion scores. Regulatory T cells and dendritic cell subsets showed isolate-dependent associations, associating with reduced CFU and lesion score in MAC104 infection only.

To identify immune correlates of protection or susceptibility across different MAC isolates, we performed Pearson correlation analysis between lung CFU, lesion score and the frequency of immune cell subsets obtained by multiparameter flow cytometry at week 16 post-infection (Fig. S2). The top seven positively and negatively correlated subsets for each isolate (MAC101, MAC104 and MAC2285R) are summarised in Fig. 5. Across all three isolates, several adaptive immune subsets – particularly activated CD4^+^ and CD8^+^ T cells – consistently exhibited correlations with increase in lung CFU and lesion scores. In MAC101 infections, effector CD8^+^ T cells (CD3^+^CD4^−^CD8^+^CD44^+^CD62L^−^) and CD69^+^ activated T cells showed strong positive associations with bacterial burden and lesion severity, suggesting that increased adaptive responses reflect ongoing infection and inflammation, potentially contributing to immunopathology. Similar trends were observed in MAC2285R infections, in which CD4^+^ effector T cells (CD3^+^CD4^+^CD8^−^CD44^+^CD62L^−^) and KLRG1^+^ natural killer cell subsets positively correlated with CFU and lesion scores, implicating heightened effector activation with increasing disease severity.

In contrast, myeloid populations – specifically, CD11b^+^Ly6G^+^ neutrophils and CD14^+^Ly6C^+^MHC-II^+^ monocytes – were consistently significantly correlated with decreased lung CFU and lesion scores across all isolates, suggesting a protective role. These populations were most enriched in animals with lower bacterial burden and reduced histopathological damage, implying their potential role in controlling infection and limiting tissue injury.

Interestingly, Tregs (CD3^+^CD4^+^CD8^−^CD25^+^Foxp3^+^) and dendritic cell subsets (CD11b^int^/highCD11c^+^) showed isolate-dependent associations. In MAC104 infections, these populations were associated with reduced CFU and lesion scores, suggesting that they contribute to immune regulation and reduced immunopathology. However, such patterns were less evident in MAC101 and MAC2285R infections.

### Chronic *M. avium* lung disease causes quantifiable evidence of increased respiratory effort

**Fig. 6.**
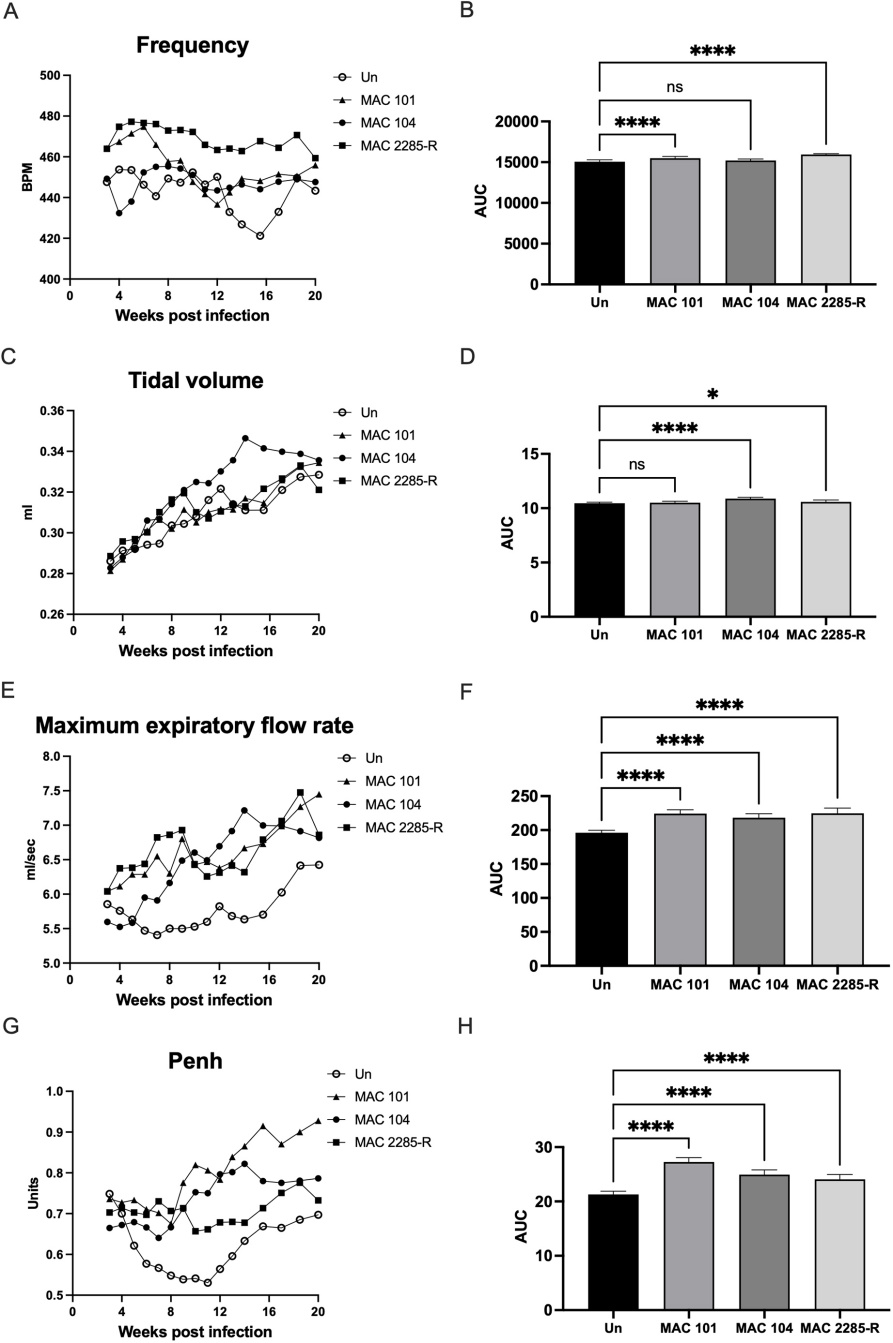
**Comparative whole-body plethysmography on BALB/c mice with chronic MAC infection.** (A-H) Mice infected with high-dose inoculum MAC2285R exhibited increasing respiratory effort over 20 weeks, as evidenced by significantly higher respiratory rate (A,B), tidal volume (C,D) and expiratory flow rate (E,F) (*P*<0.05 for all); MAC101- and MAC104-infected mice displayed similar, although less pronounced, changes in these parameters, but enhanced pause (Penh) was higher in these groups (G,H). Data points displayed as three-point moving mean for time-course graphs (with error bars removed for clarity). Data displayed as mean±s.d. for area under the curve (AUC) graphs and analysed by one-way ANOVA with Tukey's multiple comparison test using GraphPad Prism v.10. Data are representative of one study with *n*=4. *P<0.05, ****P<0.0001; n.s., not significant. BPM, beats/min; Un, uninfected.

Whole-body plethysmography (WBP) was performed weekly on BALB/c mice infected with MAC101, MAC104 or MAC2285R (*n*=4 per group) (Fig. 6). *M. avium* infection was associated with increased maximum expiratory flow rate and enhanced pause (Penh; a surrogate measure of bronchoconstriction) compared to uninfected mice, regardless of MAC isolate (*P*<0.0001 for all). Infected mice also demonstrated modest increases in respiratory frequency (MAC101 and MAC2285R) and tidal volume (MAC104 and MAC2285R). Penh was highest in mice with MAC101 infection (by ∼25%, *P*<0.0001), suggesting a greater degree of airway resistance in these mice. In addition, inspiration time and end-inspiration pause was not significantly changed by MAC infection, but all infected mice exhibited reduced expiratory time and end expiratory pause, regardless of MAC isolate (*P*<0.0001 for all) (Fig. S3). Taken together, these data suggest that chronic *M. avium* infection causes measurable increases in respiratory effort and airway resistance over time.

## DISCUSSION

Many host-related factors that contribute to MAC-PD susceptibility and severity have been uncovered (Lake et al., 2016), but this study focuses on the contribution of bacterial factors to immunopathological outcomes. Clinical MAC-PD presents in a heterogeneous spectrum of bacterial burden and associated lung pathology, comprising necrotising and non-necrotising granulomatous inflammation (Choi et al., 2021; Fujita et al., 2003). Drug distribution in the lung is affected by granulomatous inflammation, with reduced penetration observed through the fibrous cuff of necrotising granulomas, in which mycobacteria remain viable (Irwin et al., 2016; Kokesch-Himmelreich et al., 2022). Disease severity also correlates with impaired respiratory function, typically resembling obstructive lung disease with reduced force expiratory volume in 1 s, forced vital capacity and diffusing capacity for carbon monoxide (Park et al., 2023). Animal models of MAC-PD that replicate the spectrum of human pathology and respiratory function offer better prospects of optimising novel treatment strategies at the preclinical stage of development.

However, animal models of chronic MAC-PD remain undercharacterised with variable reproducibility (Shaw et al., 2025; Verma et al., 2019). MAC isolate-dependent variations in macrophage response during infection has been described previously (Lee et al., 2023), but very few comparative studies have been performed to determine differences between animal strains in MAC-PD: Andréjak et al. (2015) reported proliferative lung infection over 12 weeks in BALB/c, C57BL/6, nude and beige mice following aerogenic infection with MAC101. Between the immunocompetent animal strains (preferred for closer modelling of human disease), BALB/c mice demonstrated greater susceptibility to MAC infection and response to treatment than C57BL6 mice. Gross examination of mice from both strains found multiple large inflammatory lesions, but histopathological analysis was not performed in that study. A more recent study infected BALB/c mice with MAC101 and MAC104, demonstrating a difference in net increase in lung bacterial burden over 12 weeks between the isolates (by 0.95 log_10_ CFU and 1.5 log_10_ CFU, respectively) (Binayak et al., 2025). This concurs with our findings, in which there was net increase in both MAC101 and MAC104 lung bacterial burden over the first 12 weeks, with slightly more in the MAC104-infected mice. Both isolates generated gross pathology observable in lungs at week 8 post-infection, but no lesion quantification was performed. Another study examined the response of C57BL/6 mice infected with either MAC101 or MAC104, in relation to biofilm production and response to antimicrobials, over 12 weeks (Offman et al., 2024). MAC104 infection lung burden increased by ∼1 logfold over 12 weeks (from ∼5 log_10_ CFU to 6 log_10_ CFU, respectively), whereas only 0.5 logfold increase in CFU was seen in MAC101 infection, although the baseline infection appeared to be higher (∼7.5 log_10_ CFU). Histopathological findings were presented as descriptions of inflammatory cell infiltration, congestion and presence of granulomas, but these were not quantified. Our study offers additional knowledge by extending the timeframe of chronic infection (from 12 to 20 weeks), in a three-way MAC isolate comparison and quantifying differences in histopathology, flow cytometry and plethysmography.

MAC101 is the standard bacterial reference isolate for antimicrobial susceptibility testing, and it is well established that aerogenic MAC101 generates a proliferative lung infection in BALB/c mice (Froment et al., 2024; Lanoix et al., 2020). However, to date, characterisation of lung pathology in MAC101 infection has been modest, and our data confirm that granulomatous disease quantifiably increases over 20 weeks’ infection. We also included comparison with other isolates, MAC104 and MAC2285R, demonstrating an isolate-dependent divergence in bacterial burden and granulomatous inflammation in the later stages of infection (weeks 16-20). Surprisingly, the respiratory clinical isolate MAC2285R did not cause a proliferative lung infection, in contrast to that reported in other mouse strains (Cotroneo et al., 2024; Verma et al., 2019). This may represent negligible replication of MAC2285R *in vivo*, or a balance of bacterial replication and bacterial death.

Our findings are consistent with others who have discovered a variety of microbiological and inflammatory responses in BALB/c mice infected with clinical MAC isolates (Koji et al., 2022; Lee et al., 2023). One comparative study of nine different clinical isolates in intranasally infected BALB/c mice reported a range of proliferating and non-proliferating infections, with only virulent isolates producing non-necrotising lung granulomas after 12 weeks (Koji et al., 2022). A similar range of findings has been reported with clinical MAC-infected BALB/c mice at other centres, encompassing mild, self-resolving inflammation (González-Pérez et al., 2013) through to mature granulomatous disease (Masayuki et al., 2021; Matsumura et al., 2017; Matsuyama et al., 2014). To date, only two studies have quantified histological changes in the BALB/c mouse in response to treatment, both from the same authors: in mice infected with the clinical isolate MAC SMC #7, small, non-necrotic granulomatous inflammation occupied ∼20% of the lung tissue after 8 weeks (Lee et al., 2021) and up to 60% of the lung tissue after 12 weeks (Lee et al., 2023). This was reduced by clarithromycin monotherapy therapy from weeks 8 to 12 to ∼40% (*P*<0.05) (Lee et al., 2023) and by combination therapy to ∼10% (*P*<0.05) (Lee et al., 2021). Our data build on these studies to provide a baseline for expected degrees of granulomatous inflammation in BALB/c mice infected with three different MAC isolates over an extended chronic infection.

The immune responses observed in MAC infections provide critical insights into host–pathogen interactions, revealing distinct isolate-specific dynamics with significant scientific and clinical implications. Myeloid cell responses were most pronounced in MAC2285R-infected lungs, characterised by elevated levels of neutrophils, macrophages and monocytes, which are crucial for phagocytosis and early pathogen control. However, this robust inflammatory response may contribute to tissue damage if not properly regulated. In contrast, MAC101-infected lungs exhibited the highest levels of dendritic cells, highlighting their pivotal role in antigen presentation and the initiation of adaptive immunity. These findings suggest that MAC2285R triggers an aggressive inflammatory response that controls bacteria burden very early at expenses of increased respiratory effort, whereas MAC101 elicits a more balanced activation of innate immunity. One possible explanation for this lies in the rough phenotype of MAC2285R versus the smooth phenotype of MAC101 and MAC104. Rough isolates of MAC typically have missing or modified glycopeptidolipids antigens in their cell wall, which provoke macrophage activation and a stronger innate immune response (Belisle et al., 1993; Bhatnagar and Schorey, 2006).

The adaptive immune responses further highlight the variability among MAC isolates. MAC104 infection resulted in the highest levels of activated CD8^+^ T cells, emphasising the critical role of cytotoxic T cells in controlling intracellular pathogens. Indeed, after initial proliferation, MAC104 burden fell between weeks 12 and 20. By comparison, MAC101 and MAC2285R triggered similar, but slightly lower, levels of activation. Notably, MAC101 infection was associated with the highest levels of central memory T cells (both CD8^+^ and CD4^+^), possibly related to a chronic, high-burden infection, but also indicating its potential to prime long-term immune protection and sustain pathogen control. This suggests that MAC101 could provide valuable insights into vaccine design, particularly for eliciting durable immunity. MAC104-infected lungs exhibited significantly elevated levels of Tregs, which are essential for controlling excessive inflammation but may also suppress protective immune responses. This immunoregulatory environment in MAC104 infections could contribute to chronic infection, highlighting the need for interventions that modulate Treg activity to restore effective immunity without exacerbating inflammation. Overall, these findings demonstrate the isolate-specific immunological landscapes of MAC infections: MAC2285R induces a potent, but potentially tissue-damaging, myeloid response; MAC101 balances immune activation and memory formation; and MAC104 emphasises immune regulation, potentially at the cost of pathogen clearance.

The plethysmography data provided a novel aspect to MAC-PD modelling in the BALB/c mouse. We have previously reported WBP changes in βENaC transgenic mice and C57BL/6 wild-type controls with *Mycobacterium abscessus* pulmonary infection (Pearce et al., 2024), as well as in C3HeB/FeJ mice with MAC-PD with the same three isolates investigated in this study (Shaw et al., 2025). In βENaC transgenic mice, impaired respiratory function correlated with extensive histopathological changes over 60 days, with evidence of decompensating respiratory function towards the study end point (Pearce et al., 2024). By comparison, C3HeB/FeJ mice with MAC-PD exhibited a milder impairment of respiratory function, characterised by increased breathing rate, tidal volume and expiratory flow rates (Shaw et al., 2025). MAC2285R was associated with the strongest effect on respiratory function in the C3HeB/FeJ mouse, followed by MAC104, whereas MAC101 caused minimal change in breathing patterns. In this study of BALB/c mice, MAC2285R again generated the greatest rise in respiratory frequency, tidal volume and expiratory flow rate. However, Penh was consistently higher for MAC101 (by ∼25%), followed by MAC104 and MAC2285R. Given that Penh is a surrogate, composite measure of bronchoconstriction, this ostensibly correlates with the degree of histopathological and inflammatory lung disease. As previously suggested, these findings give greater warrant to using longitudinal WBP in preclinical models of MAC-PD to measure response to infection and therapies (Shaw et al., 2025).

Finally, we sought to deduce associations between MAC isolates, lung bacterial burden and pathology through correlation analysis at week 16 post-infection. Higher infiltrates of myeloid cells (particularly CD11b^+^Ly6G^+^ neutrophils and CD14^+^Ly6C^+^MHC-II^+^ monocytes) correlated negatively with both bacterial burden and lesion scoring. By contrast, a higher proportion of activated CD4^+^, CD8^+^ and CD69^+^ T cells correlated with higher bacterial burden and lesion scoring. There are several possible, and potentially overlapping, explanations for these findings. They suggest that a strong innate immune response is critical for achieving bacterial control, and that this is largely sufficient to prevent proliferation of less-virulent MAC isolates such as MAC2285R in this setting. Interestingly, MAC2285R-infected mice exhibited increased respiratory effort despite the absence of proliferative lung infection or significant histological injury over 20 weeks, possibly due to the cytokine response induced by the myeloid cell infiltrate. A stronger adaptive immune response, such as that seen with MAC101, may reflect failure to control a virulent isolate with innate immunity, triggering an infiltrate of lymphocytes and granulomatous inflammatory response to contain the MAC. Where the host is then able to achieve reduction in lung bacterial burden, as seen in MAC104-infected mice, a population of Tregs (CD3^+^CD4^+^CD8^−^CD25^+^Foxp3^+^) and dendritic cell subsets (CD11b^int^CD11c^+^) presents to promote inflammatory resolution and limitation of tissue injury. In keeping with this, the lesion scores and surrogate measure of increased respiratory effort (respiratory frequency, tidal volume, maximum expiratory flow rate and Penh) appear to fall or remain stable from weeks 16 to 20 in mice with MAC104 infection, whereas they continue to increase in those with MAC101 infection. Thus, in summary, MAC2285 caused a low virulence infection, generating a strong myeloid response without an accompanying lymphocyte infiltrate, resulting in increased respiratory effort but not significant granulomatous tissue damage; MAC101 caused a virulent, proliferative infection over 20 weeks that triggered a strong adaptive response, driving progressive granulomatous inflammation and increasing airway resistance; and MAC104 initially caused a proliferative lung infection, which came under control by week 16, characterised by Treg response, static pathology and stabilising respiratory function.

There were several limitations to this study. We used a high infecting inoculum of each isolate of MAC. We and others have demonstrated that the infecting dose of inoculum influences the natural course of infection, with lower inocula (e.g. 1000 CFU) generating a more proliferative infection (Shaw et al., 2025), which is likely to affect the pathology and cellular dynamics and, therefore, impact the clinical relevance of the model. Another limitation was the absence of an uninfected group for the flow cytometry. Although we are able to analyse differences between MAC isolates in the cellular response induced, an uninfected group would help determine the absolute change in cell types induced by infection. Third, the virulence of MAC isolates is susceptible to change in the laboratory and between host species. Rough morphotypes of MAC, such as MAC2285R, are associated with clinically aggressive disease, yet MAC2285R did not generate a virulent infection in this study. This may be due to loss of virulence factors during laboratory culture (e.g. from the presence of Tween 80 in the broth) or intrinsic resistance in the BALB/c mouse.

In conclusion, it was previously shown there are MAC isolate-dependent differences in the natural course of chronic lung infection and histological injury. For the first time, we have quantified these differences and added novel insights into lung immune cell profiling and WBP in prolonged murine infection (20 weeks). By performing correlation analysis on lung bacterial burden, pathology and immune cell dynamics in MAC-PD caused by three clinically relevant MAC isolates, this study adds valuable insight for investigations of emerging antimicrobial and host-directed therapies.

## MATERIALS AND METHODS

### Bacteria

Bacterial culture *M. avium* subsp. hominissuis 101 (MAC101) was isolated in 1983 from human blood, and it is also referred to as Chester MAC or ATCC 700898 (Saunders et al., 2002). This whole-genome sequenced bacterial isolate is the standard isolate for MAC susceptibility testing. *M. avium* 104 (MAC104) was isolated from an adult patient with AIDS in Southern California in 1983 (Saunders et al., 2002). The *M. avium* 2285R (MAC2285R) isolate with a rough colony morphology and positive for biofilm formation was obtained from a pulmonary MAC patient with a fibrocavitary form of disease (gift from Drs Stephen Holland and Kenneth Olivier, National Institute of Allergy and Infectious Diseases, Bethesda, MD, USA) (Verma et al., 2019). Stock cultures for each isolate were grown at 37°C in Middlebrook 7H9 liquid medium (HiMedia, M198-500G) supplemented with 10% oleic albumin dextrose catalase, 0.5% glycerol and 0.5% Tween 80. Cultures were shaken for 7-14 days to reach exponential growth phase and removed at optical density at 600 nm (OD_600_) 0.6-0.8 for experimental use, as previously described (Gonzalez-Juarrero et al., 2021). Culture was passed through a 26.5 G needle 15-20 times prior to dilution to minimise clumping.

### Animal infection

Female BALB/c mice were purchased from The Jackson Laboratory at 6-8 weeks of age. All protocols and use of these animals were approved by the Institutional Animal Care and Use Committee (IACUC) at Colorado State University. Two doses of a bacterial suspension in saline were delivered intratracheally as an intrapulmonary spray instillation to each animal using a high-pressure syringe device (PennCentury), for a targeted dose of 1×10^5^ CFU/lung as previously described (Gonzalez-Juarrero et al., 2021). To confirm the actual bacterial deposition in the lungs, mice (*n*=3) were sacrificed within 24 h after instillation, and their lungs were prepared for bacterial burden quantification. Lungs, spleen and liver were harvested from mice (*n*=3-4) at 4-week timepoints until week 20 post-infection to measure bacterial burden and lung histopathological scoring. In an independent second study, animals were sacrificed at weeks 4, 16 and 20 only, and data were combined with those from the first study for analysis. WBP was performed in the first study only. Flow cytometry was performed on lung cells isolated at week 16 post-infection in the second study only. Animals were randomised by cage to these timepoints, prior to infection. Investigators were aware of experimental groups at all stages.

### Bacterial burden enumeration

Viable bacteria burden was determined by homogenising lungs, spleen and liver in 4000 μl sterile PBS using a Precellys Tissue Homogenizer (Precellys Lysing Kit, 220325-830). Thereafter, 100 μl of serial fivefold dilutions of each homogenate were plated onto Middlebrook 7H11 agar plates (Millipore, M0428-500G) containing carbenicillin (Sigma-Aldrich, C1389-1G) and cycloheximide (GoldBio, C-930-10) and subsequently cultured for 14 days at 37°C until CFU were visible and could be enumerated, as previously described (Shaw et al., 2025). The limit of detection was determined to be the lowest CFU detectable in the sample and calculated as follows: 1 CFU in 0.1 ml homogenate plated neat from 4 ml total sample=40 CFU/ml.

### Histopathology and lesion scoring

The lungs were fixed in 4% paraformaldehyde (PFA) for 48 h then embedded in paraffin for histopathology, as previously described (Shaw et al., 2025). Sections from formalin-fixed and paraffin-embedded tissues were cut at 5 µm, stained with H&E or Ziehl–Neelson and scanned at 40× magnification using a multispectral automated PhenoImager (Akoya Biosciences) for histopathological evaluation and detection of AFB. The extent of lung lesion burden was quantified in digital images in a masked manner using open-source QuPath software for image analysis as described previously (Dutt et al., 2022). For each tissue section, a region of interest was generated at low magnification with a custom tissue detecting algorithm using decision forest training and classification to differentiate tissue versus background based on colour and area.

### Analysis of immune cell populations via flow cytometry

A single-cell suspension of lung from BALB/c mice infected with different MAC isolates was prepared as described previously (Dutt et al., 2022). Cells were counted using a Countess 3 cell counter (Invitrogen), and 1×10^6^ cells were added to a 96-well V-bottom plate (Greiner Bio-One, 651-180). Cells were incubated with Brefeldin for 5 h at 37°C in a CO_2_ incubator. After 5 h, cells were taken out of the incubator, washed with 1× PBS, and viability staining was performed by incubating cells with 100 µl Zombie NIR (BioLegend, 423106) viability dye (1:2000 dilution prepared in PBS) for 15 min in the dark. Cells were washed twice with FACS buffer (1× PBS+2% FBS+0.05% sodium azide), and Fc receptors were blocked using a 1:200 dilution of anti-mouse CD16/32 antibody (BioLegend, 156604). Cells were stained with surface antibody cocktails for myeloid cells and for T cells with predetermined optimal concentrations of specific antibodies (Tables S1 and S2, respectively) for 30 min in the dark at 4°C. Cells were washed with FACS buffer to remove excess antibody, and fixed and permeabilised by incubating cells in 1× fix/perm buffer (Foxp3/Transcription Factor Staining Buffer, Ebiosciences, 00-5523-00) for 1 h at room temperature in the dark. Cells were centrifuged, washed using 1× permeabilisation buffer, and finally stained with intracellular antibody cocktails for myeloid and T-cell panels (Tables S3 and S4, respectively). Cells were washed using 1× permeabilisation buffer and resuspended in 100 μl FACS buffer. Samples were acquired using a Cytek AuroraTM 4-Laser spectral flow cytometer, and 100,000 events were recorded. Gating strategies for myeloid and T cells are depicted in Figs S4 and S5, respectively. Data were analysed in FlowJo software (BD Biosciences).

### Whole-body plethysmography

WBP was performed as previously described (Pearce et al., 2024; Shaw et al., 2025). WBP was performed weekly for 20 weeks on both uninfected and infected BALB/c mice (*n*=4). As breathing patterns are susceptible to environmental stimuli, mice were equilibrated within individual WBP chambers of a Buxco FinePointe Series Whole Body Plethysmograph (DSI Buxco respiratory solutions, Data Sciences International) for 10 min before 10 min data acquisition. Data were collected at the same time of day, within a 4-h window. The investigator would leave the room during data acquisition and observe through a window. Surrogate measures of respiratory effort and timing were taken, including respiratory frequency, tidal volume per breath, maximum inspiratory flow rate, maximum expiratory flow rate, inspiration time, end-inspiration pause, expiration time and end-expiration pause. Penh, a composite measure commonly used in studying airway resistance in rodents, was included to infer bronchoconstriction, as previously described (DeLorme and Moss, 2002; Julander et al., 2014; Pearce et al., 2024). A three-point moving average for each parameter was calculated from the mean of the measured point and the preceding two points (Pearce et al., 2024). Area under the curve was calculated for each parameter over the study period for comparative analysis between groups. Data reports were generated in the accompanying FinePointe software and exported to GraphPad Prism v.10 for analysis.

### Statistical analysis

Bacterial burden data were expressed as CFU, which were log_10_ transformed and analysed using GraphPad Prism v.10. Statistical analysis was performed using one-way ANOVA with Tukey's multiple comparison test (for comparing data between different groups) or unpaired two-tailed *t*-tests (for comparing data from different time points within the same group). For immune cell responses, data were plotted using ggplot2 in R, and statistical significance was evaluated using the R package rstatix. Correlation analysis was performed using Pearson correlation analysis.

### Ethics statement

Animal studies were approved by the Association for Assessment and Accreditation of Laboratory Animal Care (AAALAC)/Colorado State IACUC. The experiments were conducted in accordance with local legislation and institutional requirements.

## Supplementary Material

10.1242/dmm.052671_sup1 Supplementary information
