# Supplementary material for: Immunopathological outcomes are isolate dependent in chronic Mycobacterium avium complex pulmonary disease
Source: Dis Model Mech. 2026 Jan 30;19(1):dmm052671. doi: 10.1242/dmm.052671 (PMC12893035; doi:10.1242/dmm.052671)
Supplement: Supplementary information [file dmm-19-052671-s1.pdf]

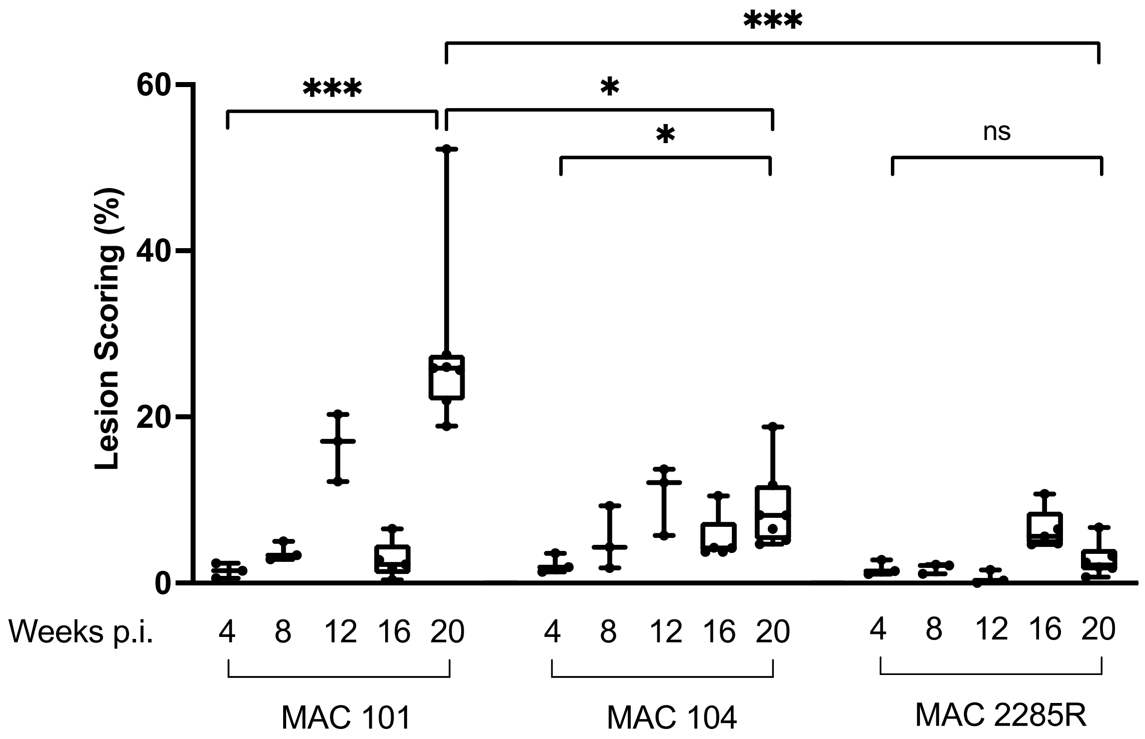

Fig. S1. Lesion scoring at 4-weekly intervals during MAC infection

Mice exhibited progressive increases in mean lesion scoring between weeks 4 and 20 post-infection when infected with MAC101 or MAC104 ($p<0.001$ and $p<0.05$ respectively). By contrast, lesion scoring remained unchanged in mice with MAC2285R infection over the study period ($p=0.34$). By week 20, MAC101 infection resulted in higher lesion scores (mean 28.3%) than MAC104 (mean 9.0%, $p<0.05$) and MAC2285R (mean 2.8%, $p<0.001$). Data combined from two independent studies, n=3-7 per time point. Data was analysed by one-way ANOVA with Turkey's multiple comparison test for comparison between MAC isolates, or by unpaired t-test for comparison between time points with the same isolate. *$p<0.05$, ***$p<0.001$, n.s. not significant.

Disease Models & Mechanisms • Supplementary information

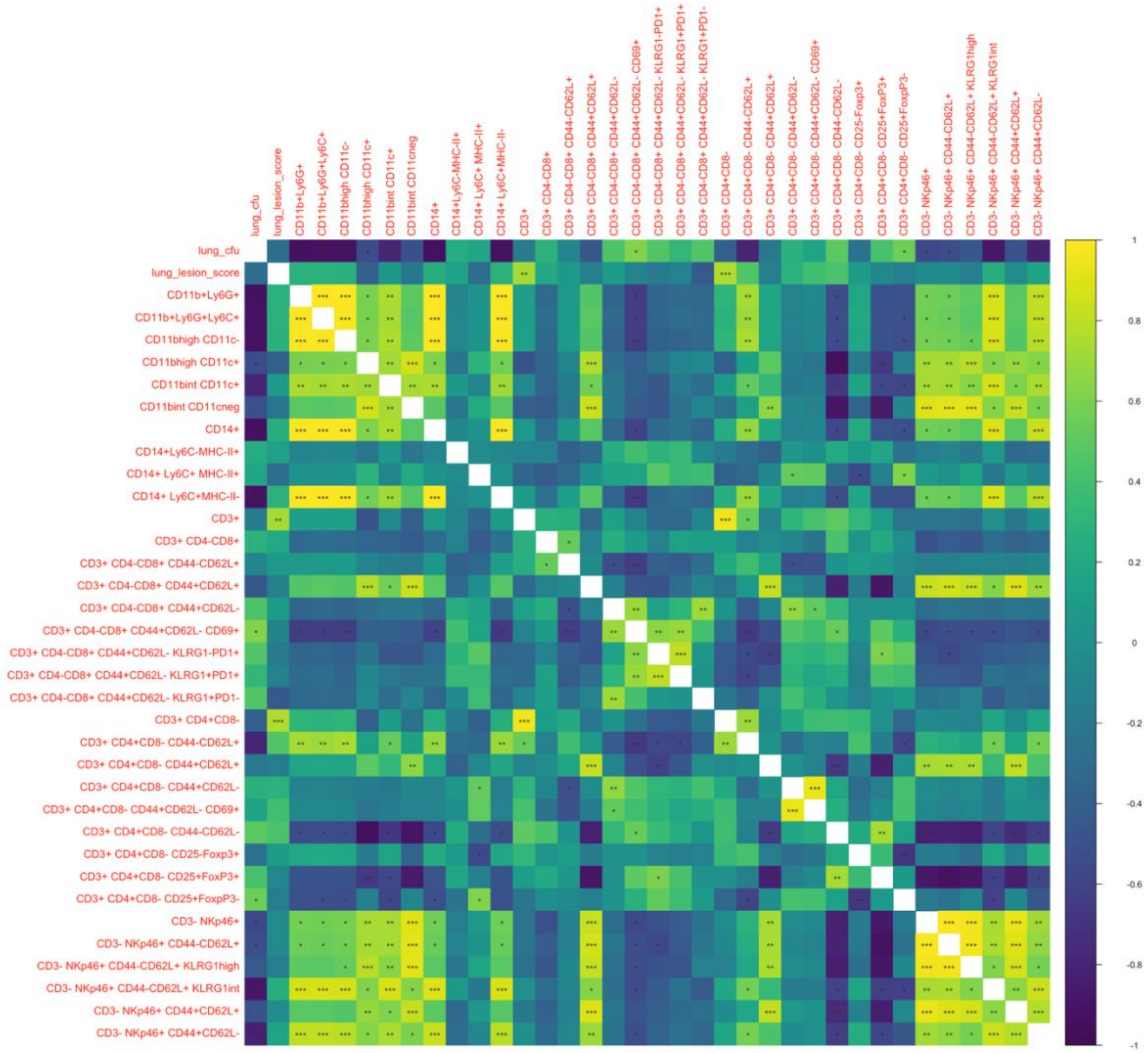

Fig. S2. Correlation of lung immune cell subsets with bacterial burden

To identify immune correlates of protection or susceptibility across different MAC isolates, we performed Pearson correlation analysis between lung CFU, lesion score, and the frequency of immune cell subsets obtained by multiparameter flow cytometry at week 16 post-infection. * $p<0.05$, ** $p<0.01$, *** $p<0.001$

Disease Models & Mechanisms • Supplementary information

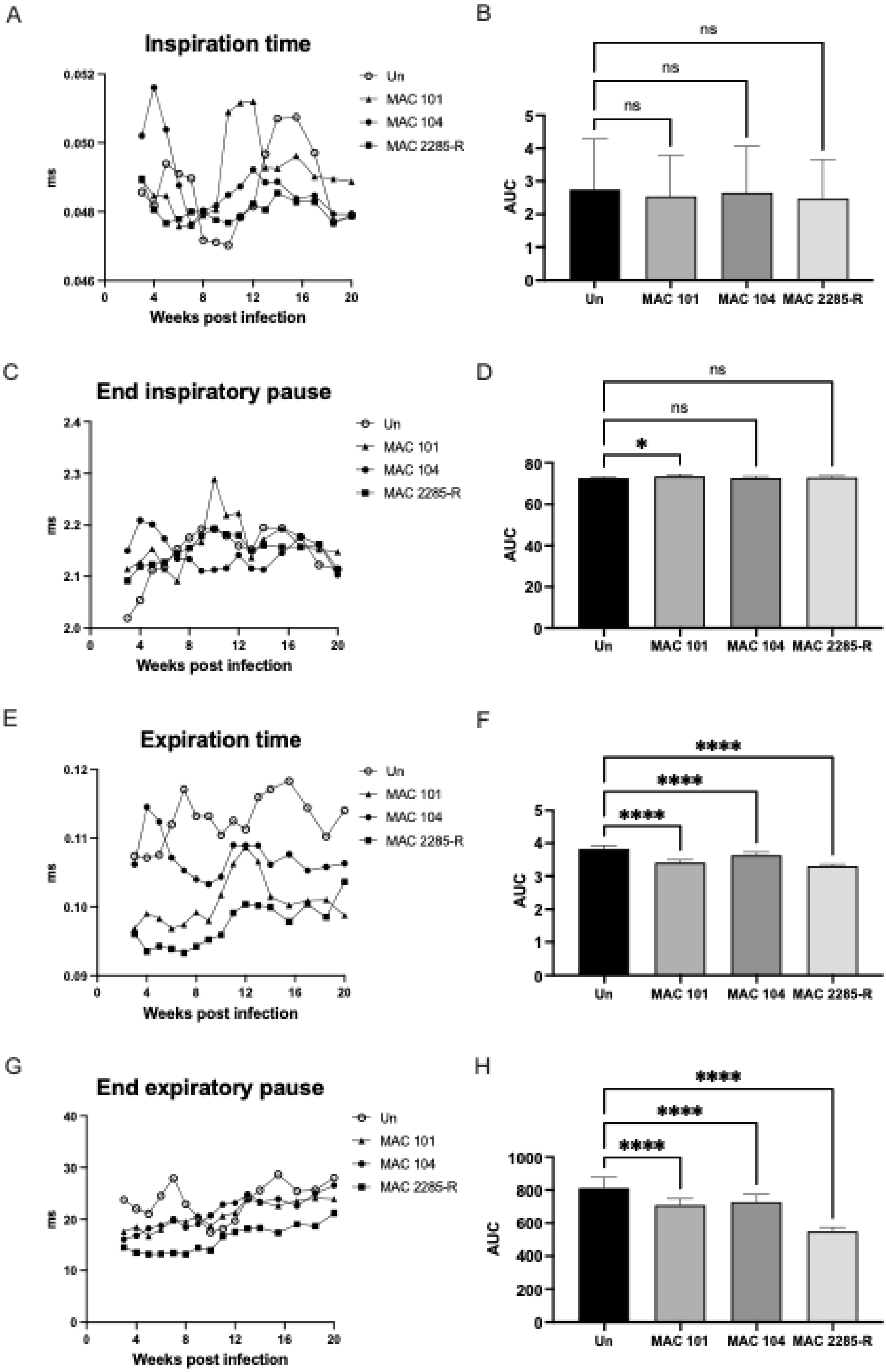

A
Inspiration time
ms
Weeks post infection
Un
MAC 101
MAC 104
MAC 2285-R
B
AUC
ns
ns
ns
Un
MAC 101
MAC 104
MAC 2285-R
C
End inspiratory pause
ms
Weeks post infection
D
AUC
ns
ns
*
E
Expiration time
ms
Weeks post infection
F
AUC
****
****
****
G
End expiratory pause
ms
Weeks post infection
H
AUC
****
****
****

Fig. S3. Respiratory timing in mice with MAC pulmonary infection

Infection caused little change in inspiration time (E) and end-inspiration pause (F), but all infected mice exhibited reduced expiratory time (G) and end expiratory pause (H), regardless of MAC isolate ($p<0.0001$ for all). Data points displayed as 3-point moving mean for time-course graphs (with error bars removed for clarity). Data displayed mean+SD for AUC graphs and analysed by one-way ANOVA with Turkey's multiple comparison test using Prism Graphpad v.10. Data representative of one study with n=4. *$p<0.05$, ****$p<0.0001$, n.s. not significant. ▲ MAC 101; ● MAC 104; ■ MAC 2285-R; O Uninfected (Un)

Disease Models & Mechanisms • Supplementary information

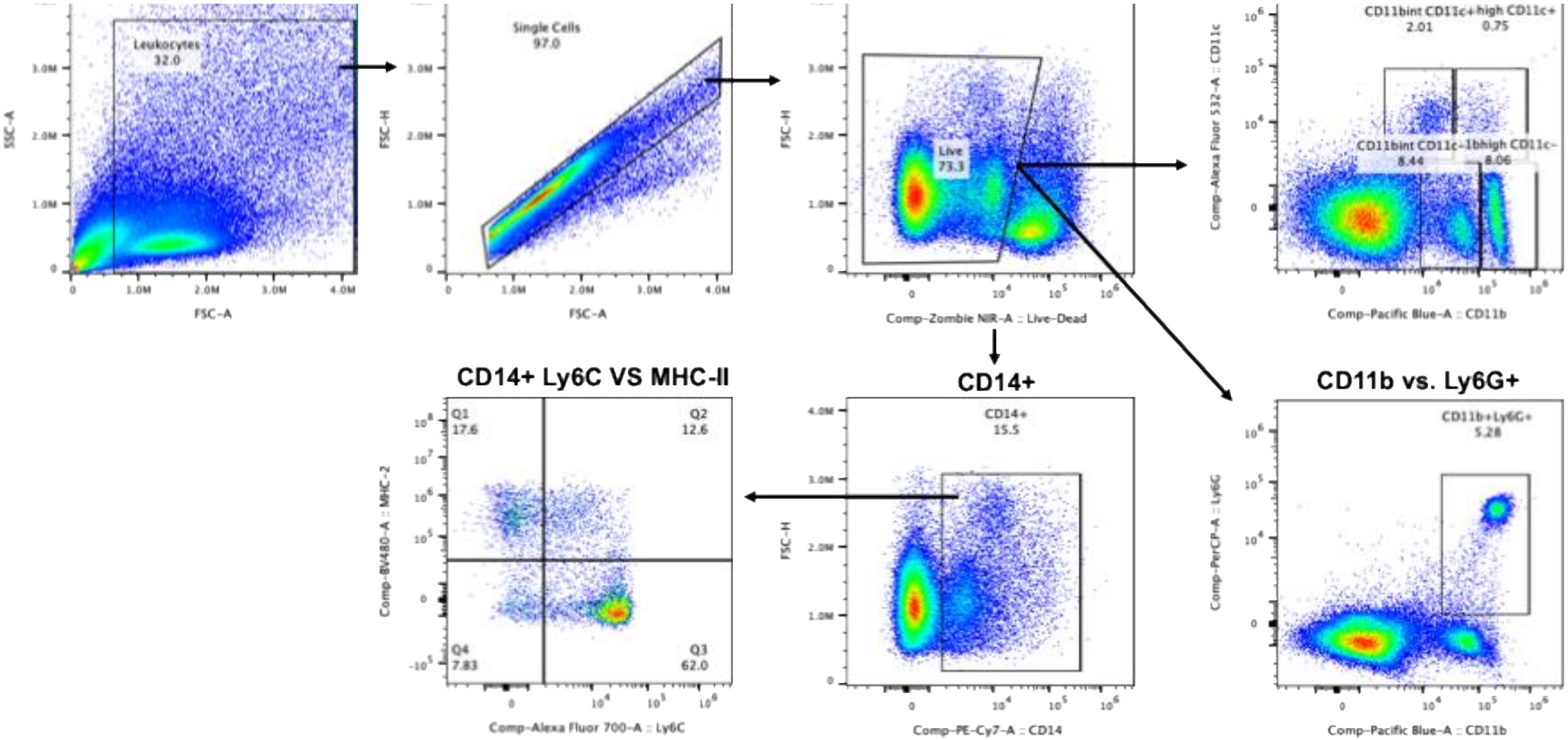

Fig. S4. Gating strategy for myeloid cells

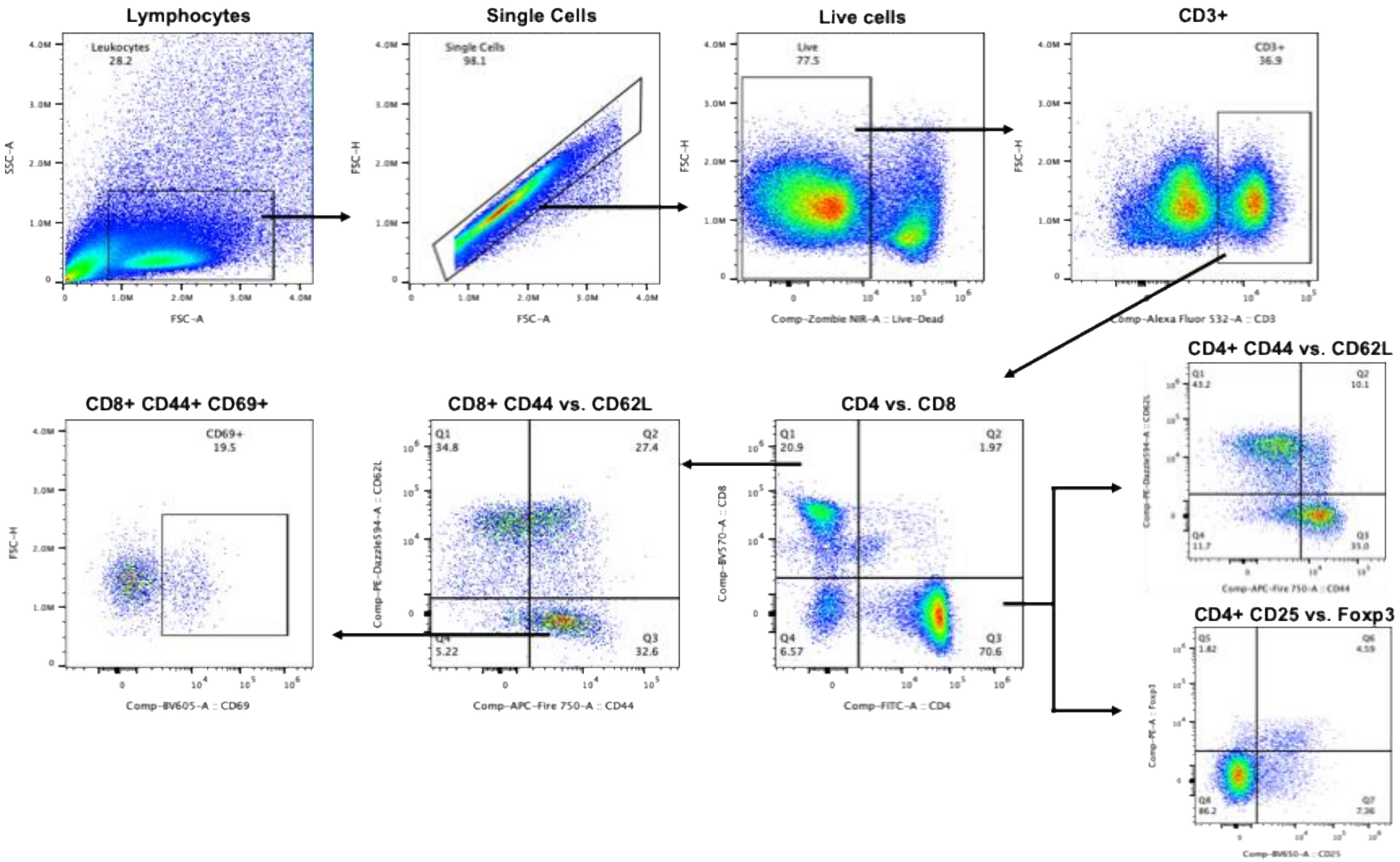

Fig. S5. gating strategy for lymphocytes

Disease Models & Mechanisms • Supplementary information

Table S1. Myeloid cell panel for surface markers

Fluorophore	Marker	Clone	Company	Catalogue #	Optimized concentration
AF-532	CD11c	M418	Invitrogen	58-0114-82	1 µg/ml
PE Dazzle 594	CD204	1F8C33	Biolegend	154718	2 µg/ml
PerCP	Ly6G	127654	Biolegend	127654	1 µg/ml
FITC	CD14	Sa-14-2	Biolegend	123308	2.5 µg/ml
PE Cy7	CD86	GL-1	Biolegend	105014	1 µg/ml
Alexa 647	Siglec F	E50-2440	BD biosciences	562680	1 µg/ml
Alexa 700	Ly6C	HK1.4	Biolegend	128024	2.5 µg/ml
Pacific Blue	CD11b	M1/70	Biolegend	101224	1 µg/ml
BV 480	MHC II	M5/114-15.2	BD biosciences	566086	1 µg/ml
BV570	CD45	30-F11	Biolegend	103136	1 µg/ml
BV605	CD68	CXCR2	BD biosciences	747814	1 µg/ml
BV 650	CD206	C868C2	Biolegend	141723	1 µg/ml
BV 711	CCR2	475301	BD biosciences	747964	1 µg/ml
BV786	CD64	X54-5/7.1	BD biosciences	741024	1 µg/ml

Table S2. T cell panel for surface markers

Fluorophore	Marker	Clone	Company	Catalogue #	Optimized concentration
FITC	CD4	GK 1.5	Biolegend	100406	2 µg/mL
Alexa fluor 532	CD3	17A2	eBiosciences	58-0032-82	2.5 µg/mL
BV480	MHC-II	M5/114-15.2	BD biosciences	566086	1 µg/mL
BV650	CD25	PC-61	Biolegend	102038	2 µg/mL
BV711	NKp46	29A1.4	Biolegend	137621	1 µg/mL
BV785	KLRG1	2F1	Biolegend	138421	1 µg/mL
BV605	CD69	H1.2F3	Biolegend	104530	2 µg/mL
PE Dazzle 594	CD62L	MEL-14	Biolegend	104448	0.8 µg/mL
PerCP-CP-efluor 710	PD1	J43	eBiosciences	46-9985-82	1 µg/mL
BV570	CD8	53-6.7	Biolegend	100740	2.5 µg/mL
APC	CD27	LG.3A10	Biolegend	124212	2 µg/mL
APC fire 750	CD44	IM7	Biolegend	103062	0.5 µg/mL

Table S3. Myeloid cell panel for intracellular markers

Fluorophore	Marker	Clone	Company	Catalogue #	Optimized concentration
PE	iNOS	W16030C	Biolegend	696806	2 µg/mL
BV421	IL-10	JES5-16E3	Biolegend	505022	4 µg/mL

Table S4. T-cell panel for intracellular markers

Fluorophore	Marker	Clone	Company	Catalogue #	Optimized concentration
PE	Foxp3	MF-14	Biolegend	126404	2 µg/mL
Pacific blue	TNFa	MP6-XT22	Biolegend	506318	4 µg/mL
PE Cy7	IFNy	XMG1.2	Biolegend	505826	1 µg/mL
BV421	Il-10	JES5-16E3	Biolegend	505022	4 µg/mL
AF700	IL-17A	TC11-18H10.1	Biolegend	506914	2.5 µg/mL
